# *Schusteria marina* sp. nov. (Acari, Oribatida, Selenoribatidae) an intertidal mite from Caribbean coasts, with remarks on taxonomy, biogeography, and ecology

**DOI:** 10.1080/01647954.2017.1348393

**Published:** 2017-07-07

**Authors:** Tobias Pfingstl, Andrea Lienhard

**Affiliations:** ^a^ Institute of Zoology, University of Graz, Graz, Austria

**Keywords:** Lesser Antilles, morphology, littoral, systematics, *Thalassozetes*, *Rhizophobates*

## Abstract

*Schusteria marina* sp. nov. is a newly discovered intertidal mite species found on the Lesser Antillean Islands of Martinique and Grenada. It represents the first record of the genus *Schusteria* within the Caribbean area. *Schusteria marina* sp. nov. is very similar to the type species, *S. littorea* from Brazil, but can be distinguished by its smaller body size, a median sternal dark sclerotized small ridge on epimeron I and a few other less pronounced differences. Both species are closely related and form a Western Atlantic *Schusteria* clade, which is characterized by the possession of a pair of faint anterior epimeral ridges. *Schusteria marina* sp. nov. occurs on different littoral substrates, e.g. mangrove roots, anthropogenic concrete structures, but seems to be associated with an intertidal red alga belonging to the genus *Bostrychia* Montagne, 1842 growing on these substrates.

http://zoobank.org/urn:lsid:zoobank.org:pub:C246533E-D108-4F2C-8577-545C059D46CE

## Introduction

The genus *Schusteria* Grandjean, 1968 belongs to Selenoribatidae, a family of exclusively intertidal oribatid mites occurring on coasts of the subtropics and tropics. Grandjean (1968) described the type species, *Schusteria littorea*, based on specimens from Brazil. A further four *Schusteria* species have been described, but all of them have been subject to taxonomic controversies.

Marshall and Pugh (2000) described *Schusteria melanomerus* and *Schusteria ugraseni*, which inhabit marine shores of southern Africa, but Karasawa and Aoki (2005) questioned this classification and transferred both species to *Rhizophobates*. Later, Pfingstl and Schuster (2012) as well as Pfingstl (2016) argued that both taxonomic transfers were unjustified and that these South African species should remain in *Schusteria*.

Karasawa and Aoki (2005) described two other species, *Schusteria nagisa* and *S. saxea*, from Japanese coasts. Pfingstl and Schuster (2012) doubted their classification, arguing that it was based on inaccurate premises. They refrained from changing the classification and suggested that the generic diagnosis of *Schusteria* should be reconsidered to solve these taxonomic problems, but the situation is still unclear.

*Schusteria* species collectively are widely distributed and occur transoceanically (Pfingstl and Schuster 2014). In the Atlantic Ocean, there are records of *S. littorea* and of an undetermined *Schusteria* species from shores of Brazil (Grandjean 1968); in the Indo-Pacific, *S. melanomerus* and *S. ugraseni* can be found on Southeast African coasts from Kenya to South Africa (Marshall and Pugh 2000; Pfingstl 2016), while *S. nagisa* and *S. saxea* occur on the Japanese Ryukyu Islands (Karasawa and Aoki 2005); and in the East Pacific, there are reports of *S. littorea* from Galápagos (Schatz 1998) and of an undetermined *Schusteria* species from El Salvador in Central America (Grandjean 1968).

In the Caribbean area, records of *Schusteria* were lacking so far, only members of the presumably closely related genus *Carinozetes* are known to be distributed on shores of several Caribbean islands (Pfingstl and Schuster 2012, 2014; Pfingstl et al. 2016). In the course of ongoing studies in the Caribbean region, specimens of a new *Schusteria* species were collected on two Antillean islands. The present article aims to provide a detailed description of this species, update and assess biogeographic patterns of the genus, and discuss its morphological properties.

## Material and methods

### Sample collection

Samples of intertidal algae were scraped off rocks or mangrove roots with a knife. Mites were extracted from the algae with a Berlese-Tullgren apparatus, then preserved and stored in absolute ethanol.

### Sample locations

1. Martinique, Sainte-Anne, Pointe Marin Beach; intertidal algae (*Bostrychia* sp.) from mangrove roots (*Rhizophora mangle*); 23 February 2016; coordinates 14°27'06.53″N 60°52'58.65″W.
2. Grenada, La Sagesse Bay; intertidal algae (*Bostrychia* sp.) from concrete structure; 27 February 2016; coordinates 12°01'26.68″N 61°40'18.27″W.

### Preparation of specimens

For investigation in transmitted light, preserved animals were embedded in Berlese mountant. Drawings were made with an Olympus BH-2 Microscope equipped with a drawing attachment. Light photographs were made with an Olympus E5 digital SLR camera attached to the same microscope.

## Description

***Schusteria marina* sp. nov.**

#### Type material

Holotype (preserved in ethanol): female, Sainte-Anne, Martinique, Pointe Marin. Deposited in the collections of the Senckenberg Museum für Naturkunde Görlitz (SMNG) (collection number. DNR 56570). Paratypes (preserved in ethanol): same locality as holotype, 4 Paratypes from same sample, deposited at the Naturhistorisches Museum Wien/NHM Vienna (1 male, NHMW 28630; 3 females, NHMW 28631), additional specimens in the collections of the Institute of Zoology, University of Graz.

#### Etymology

The specific name is derived from the Latin adjective “marina” which means belonging to the sea. It refers to the marine association of this species and additionally it refers indirectly to the type locality “Pointe Marin” on Martinique.

#### Diagnosis

Cerotegument overall finely granular. Prodorsal ridges absent. Sensilli very short. Dorsosejugal suture incomplete. Clear-spot present on anterior part of notogaster. Faint epimeral ridges present. Slight median sternal ridge present on epimeron I.

#### Description of adult

The new species shows all familial and generic traits given by Grandjean (1968).

Females (*N* = 5), length: 376–394 μm (mean 382 μm), width: 246–259 μm (mean 250 μm); males (*N* = 3), length: 357–381 μm (mean 368 μm), width: 225–252 μm (mean 240 μm).

#### Integument

Colour dark brown.

#### Prodorsum

Cerotegument finely granular. Rostrum rounded in dorsal view, slightly projecting anteroventrally in lateral view (Figure 2(a,c)). Rostrum demarcated from remainder of prodorsum by faint transverse ridge. Lamellar ridges completely absent. Rostral seta (*ro*) setiform, smooth (approx. 15 µm). Lamellar seta (*le*) setiform and smooth (approx. 12 µm), bent caudally. Interlamellar seta (*in*) setiform (approx. 12 µm), exobothridial seta (*ex*) minute. Bothridium large cup, with lateral incision. Sensillus short (approx. 23 µm), slightly curved caudally, clavate, and distally covered with fine conspicuous spines.

**Figure 1.**
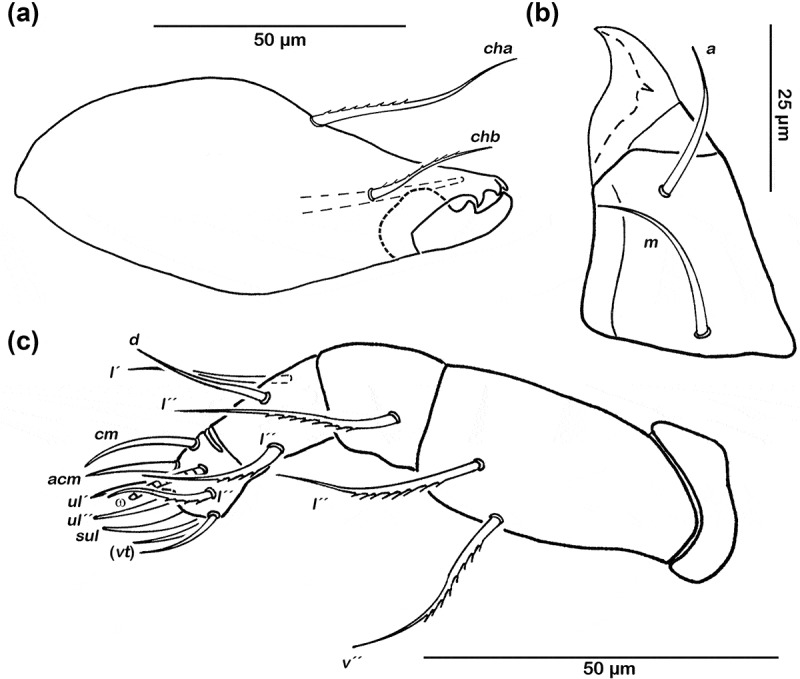
*Schusteria marina* sp. nov. adult. (a) Right chelicera, antiaxial view; (b) left rutellum ventral view; (c) left pedipalp, antiaxial view.

**Figure 2.**
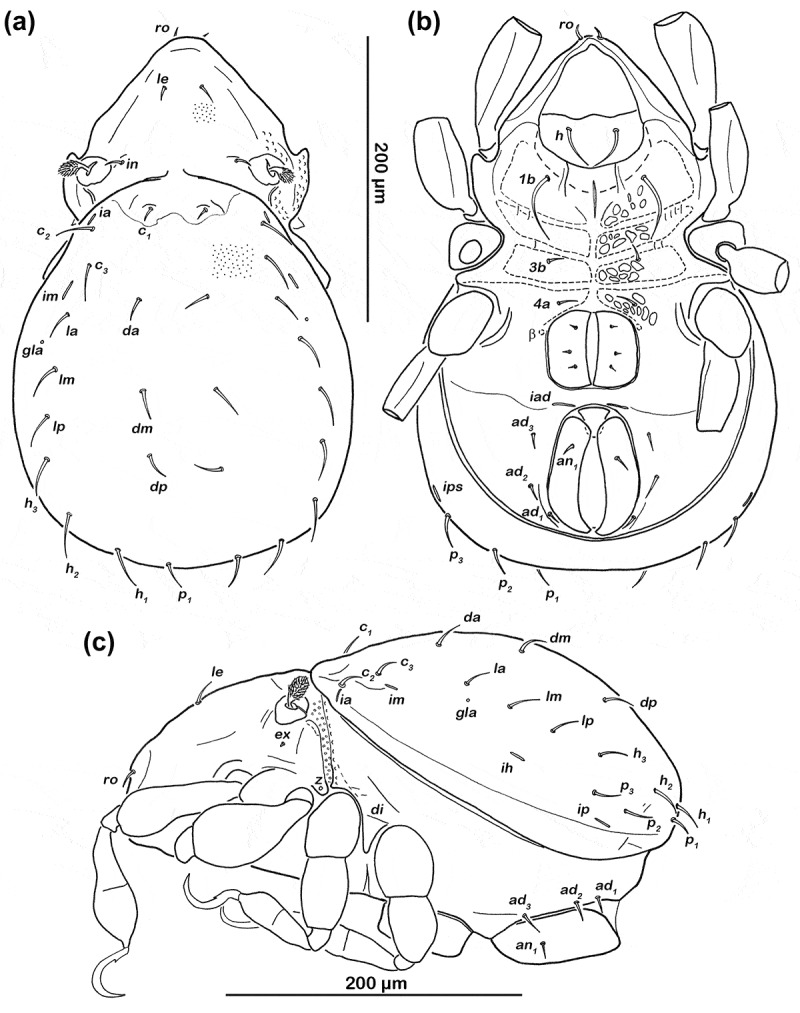
*Schusteria marina* sp. nov. adult. (a) Dorsal view, legs omitted; (b) ventral view, legs only drawn partially; (c) lateral view, tibia and tarsus II broken off.

#### Gnathosoma

Palp pentamerous, setation 0-2-1-3-8 (solenidion not included) (Figure 1(c)), lateral and ventral setae slightly pectinate. Cheliceral mobile digit darker sclerotized; distinct teeth all interlocking (Figure 1(a)). Seta *cha* (approx. 35 µm) and *chb* (approx. 25 µm) both robust and pectinate. Gena well sclerotized. Distal part of rutellum developed as thin triangular membrane, slightly curved inward with longitudinal incision (Figure 1(b)). Seta *a* and *m* long (approx. 25 µm), robust, and smooth. Mentum regular, seta *h* setiform, thin (approx. 30 µm).

#### Notogaster (Figure 2(a))

Rounded, slightly pear-shaped in dorsal view, convex in lateral view. Dorsosejugal suture incomplete, medially clearly interrupted. Cerotegument finely granular. Lighter, less sclerotized area on most anterior part of notogaster, edges variable, and poorly defined. Fifteen pairs of setiform, smooth notogastral setae (20–25 µm), *c*_1__–__3_, *da*, *dm, dp*, *la*, *lm*, *lp, h*_1__–__3_, *p*_1__–__3_. Lyrifissure *ia* lateral and slightly anterior to seta *c*_2_; *im* slightly anterior of seta *la*; *ih* lateral and anterior to *h*_3_; lyrifissure *ip* lateral to seta *p*_2_ and *ips* anterior to seta *p*_3_. Orifice of opisthonotal gland (*gla*) between setae *la* and *lm*.

#### Lateral aspect (Figure 2(c))

Cerotegument finely granular, larger granules in lateral sejugal area and in areas surrounding acetabula. Pedotectum I small, rounded. Pedotectum II absent. Discidium (*di*) developed as prominent triangular bulge. Next to bothridium small rounded cuticular ridge passing into faint lateral ridge reaching orifice of coxal gland II *z*. Opening of coxal gland on small, distinct triangular projection. Another small, but opposing triangular projection above leg II.

#### Ventral region of idiosoma (Figure 2(b))

Cerotegument finely granular. Epimeral setation 1-0-1-1, all setae setiform and smooth. Setae *1b* longest (approx. 47 µm), others short (approx. 10 µm). Internal borders of epimera I–III well visible. With a pair of parallel longitudinal small ridges reaching from camerostome to middle of epimeron I and an unpaired longitudinal darker sclerotized sternal small ridge in the middle of epimeron I. Genital and anal opening adjacent, both surrounded by slightly darker cuticle. Rounded genital plates with three pairs of equidistant fine, short filiform setae (approx. 6 µm) arranged in a longitudinal row; first two pairs closer together. Tendon β well visible as dark dot lateral to genital orifice. Aggenital setae absent. Anal valves trapezoidal. Preanal organ rectangular with rounded edges. Three pairs of short adanal setae, *ad*_1__–__3_ (approx. 15 µm). One pair of short anal setae *an*_1_ (approx. 10 µm) inserting on anterior half of anal valves. Lyrifissure *iad* transversely aligned, adjacent to anterior corners of anal opening.

##### Legs (Figure 3)

Long, broad claws with one proximoventral tooth. Cerotegument granular from trochanter to genu, finer granules from tibia to tarsus. Ventral carina present on all femora. Porose areas not discernible. Femoral dorsal setae and most tarsal setae strongly barbed. For chaetome and solenidia, see Table 1.

**Table 1.** *Schusteria marina* sp. nov. adult; leg setation, chaetome and solenidia; () = pairs of setae.

	Trochanter	Femur	Genu	Tibia	Tarsus	Chaetome	Solenidia
Leg I	*–*	*d, bv″, l'*	(*l*), σ	(*l), v'*, φ_1_, φ_2_	(*pl*), (*pv), s*, (*a*), (*u*), (*p*), (*it*), (*tc*), (*ft*), ε, ω_1_, ω_2_	0-3-2-3-18	1-2-2
Leg II	*–*	*d, bv″, l'*	(*l*), σ	(*l*), *v'*, φ	(*pv), s*, (*a*), (*u*), (*p*), (*it),(tc*), (*ft*), ω	0-3-2-3-15	1-1-1
Leg III	*v´*	*d, ev'*	*l*', σ	(*v*), φ	(*pv*), *s*, (*a*), (*u*), (*p*), (*tc*), (*ft*)	1-2-1-2-13	1-1-0
Leg IV	*v´*	*d, ev'*	*l*'	*l'*, (*v*), φ	(*pv*), *s*, (*a*), (*u*), (*p*), (*tc), ft*″	1-2-1-3-12	0-1-0

**Figure 3.**
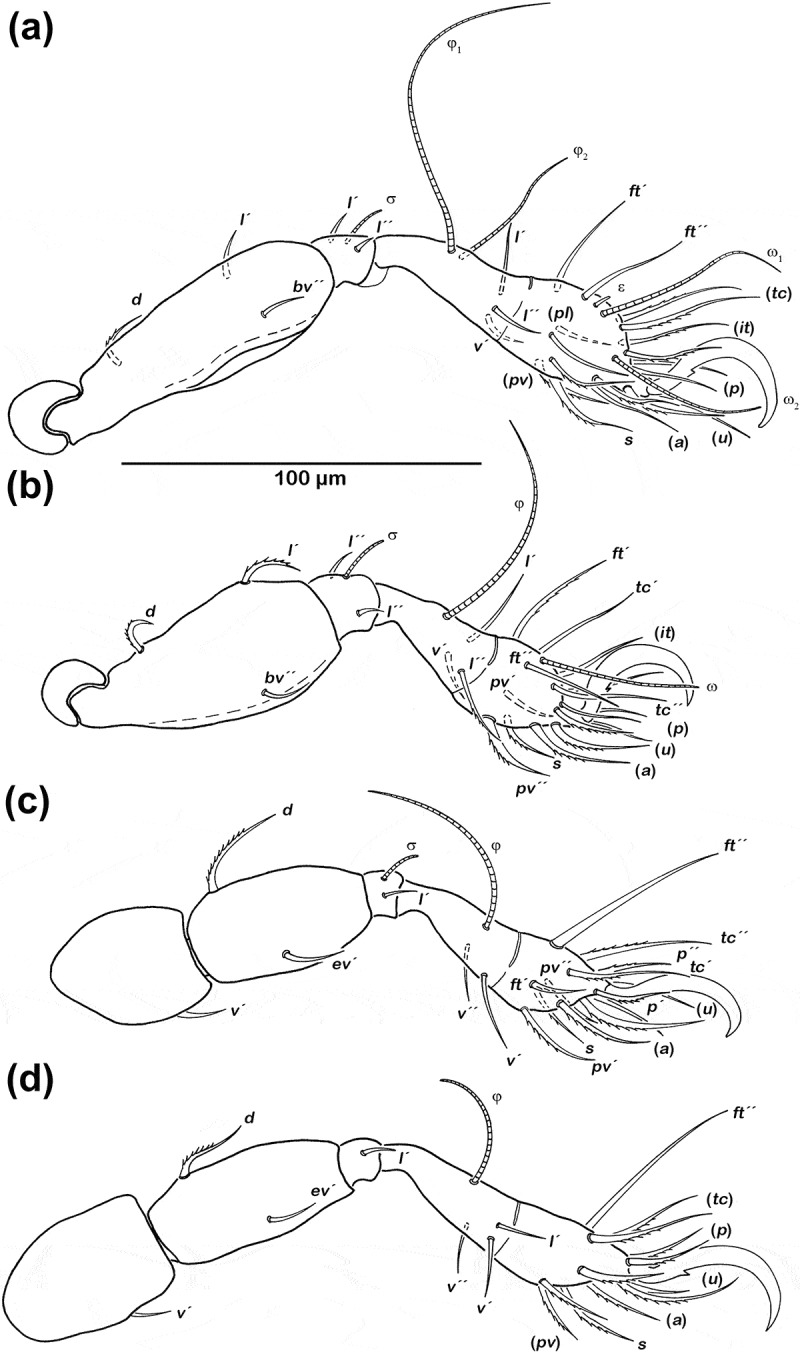
*Schusteria marina* sp. nov. adult, legs antiaxial view. (a) Right leg I; (b) right leg II; (c) left leg III; (d) left leg IV.

## Discussion

### Taxonomy

*Schusteria marina* sp. nov. shows a striking similarity to the type species, *S. littorea*. They share the same habitus, exhibit the same number and positions of body setae, and possess identical leg setation. Observed differences are not very pronounced, but include the following four traits. First, *S. marina* sp. nov. is slightly smaller than *S. littorea* (357–394 µm vs. 370–415 µm), but the slight overlap precludes discrimination on this feature alone. Second, Grandjean (1968) reported very faint and sometimes asymmetrical prodorsal ridges or impressions in *S. littorea* (labelled *cl* in his Figure 1(a), p. 119), whereas no such ridges or even traces of them could be detected in *S. marina* sp. nov. Third, *S. marina* sp. nov. shows a weakly sclerotized anterior notogastral area giving the appearance of a clear spot (see Figure 2(a)), and such a structure was not mentioned for *S. littorea* (Grandjean 1968). Fourth and most important, *S. marina* sp. nov. possesses a median unpaired longitudinal dark sclerotized sternal small ridge on epimeron I, a structure that is clearly absent in *S. littorea*.

Most of these differences are somewhat subtle and may be subject to morphological plasticity; only the dark median sternal small ridge represents a distinct character separating the two species. Grandjean did not designate types for *S. littorea* but he was an accurate observer, always providing extremely detailed information in his descriptions. Therefore, we believe that noted differences are genuine. However, based on their great morphological similarity, *S. littorea* and *S. marina* sp. nov. probably represent sister species. Their close geographical distribution is also consistent with their being sister taxa.

A critical reader may now object that found differences are just geographical variation within *S. littorea* showing a wider distribution, but based on our experience with morphometric and molecular genetic data of intertidal oribatid mites, we can state that this is highly unlikely, especially when considering the large distance of more than 3500 km between the *S. marina* sp. nov. specimens from the Lesser Antilles and the *S. littorea* specimens from Brazil.

### Geographic distribution

*Schusteria marina* sp. nov. was found in coastal habitats of Martinique and Grenada. Based on these two records, it probably also occurs on other islands of the Lesser Antilles. A wider distribution within the Caribbean area seems unlikely since comprehensive sampling by the authors in this geographic region, e.g. Curaçao, Panama, Florida, Bahamas, Jamaica, Puerto Rico, and Hispaniola (Pfingstl et al. 2016), yielded no further records of this species or even other members of this genus.

### Ecology

**Figure 4.**
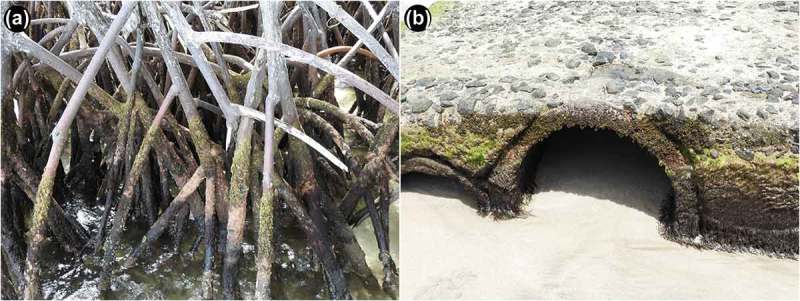
Photographs of sample locations. (A) Type locality, Martinique, Pointe Marin, mangrove roots of *Rhizophora mangle* overgrown with the intertidal alga *Bostrychia* sp.,; (B) Grenada, La Sagesse Beach, concrete block overgrown with *Bostrychia* and diverse other algae.

*Schusteria marina* sp. nov. was found in different habitats of the intertidal area which indicates a broad ecological tolerance. The population from Martinique dwelled in algae growing on mangrove roots, whereas specimens from Grenada were collected from algae growing on man-made concrete structures on a sandy beach (Figure 4). The substrate of these two locations differed markedly but in both cases the covering algae belonged to the genus *Bostrychia*. This alga serves as an important food source for intertidal mites (e.g. Pfingstl 2013), and therefore may be responsible for the occurrence of populations in apparently different habitats.

**Figure 5.**
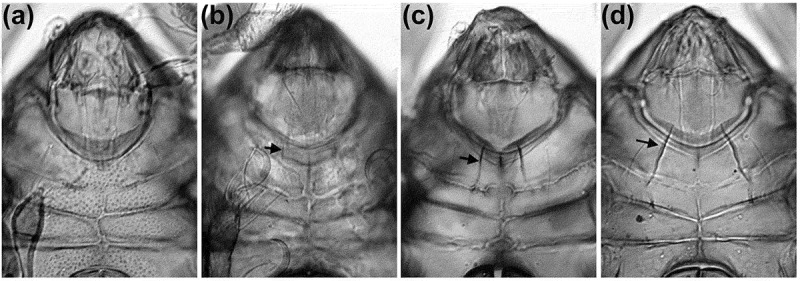
Microscopic photographs of anterior ventral region showing possible evolution of epimeral ridge from plesiomorphic absence through development as strong ventral carina. Arrowheads indicating the structure on right body side. (A) *Schusteria melanomerus* – no ridges; (B) *Schusteria marina* sp. nov. – small faint ridges; (C) *Carinozetes bermudensis* – long carinae, slightly converging; (D) *Carinozetes mangrovi* – long robust carinae, strongly converging.

*Schusteria littorea* and *S. melanomerus* are also species known to occur in various habitats: *S. littorea* was found in algae growing on intertidal rocks (Grandjean 1968) as well as in algae growing on mangroves (Pepato et al. 2010), and *S. melanomerus* was collected from roots and stems of *Avicennia* mangroves (Marshall and Pugh 2000), and from rocks overgrown with algae (Pfingstl 2016). The other *Schusteria* species were found only in a single habitat: *S. ugraseni* was sampled from empty oyster and barnacle shells (Marshall and Pugh 2000), and the Japanese *S. nagisa* and *S. saxea* were both extracted from algae growing on rocks (Karasawa and Aoki 2005). Unfortunately, none of the published include the specific type of alga collected, so ecological preferences remain unknown.

### The genus Schusteria

A comparison of certain morphological features of all described *Schusteria* species (Table 2) reveals two interesting things. First, there are several diagnostic traits that vary among species, e.g. lamellar ridges, sensillus shape, number of notogastral, adanal, and anal setae, that complicate formulation of a clear and precise diagnosis for the genus. Although Grandjean (1968) mentioned variability of certain characters, he stated that *Schusteria* always lacks lamellar ridges but *S. nagisa* and *S. saxea* clearly possess this morphological structure (Karasawa and Aoki 2005). Pfingstl and Schuster (2012) already referred to this discrepancy and mentioned the need for a revised diagnosis. We agree but refrain from giving this diagnosis because only a comprehensive revision with a concomitant molecular genetic investigation can solve these taxonomic problems in the long term.

**Table 2.** Comparison of distinguishing morphological characters of known *Schusteria* species.

	*S. littorea*	*S. marina* n. sp.	*S. melanomerus*	*S. ugraseni*	*S. nagisa*	*S. saxea*
Geographic occurrence	WA	WA	SEA	SEA	SJI	SJI
Body length (µm)	370–415	357–394	**275–305**	**331–334**	337–365	340–366
Lamellar ridges	Absent	Absent	Absent	Absent	**Present**	**Present**
Sensillus/shape/length	Clavate	Clavate	Clavate	Clavate	Lanceolate	Clavate
	**Short**	**Short**	Normal	Normal	Normal	Normal
Dorsosejugal suture	Incomplete	Incomplete	Incomplete	Incomplete	Incomplete	Complete*
Notogastral setae	15	15	14, *c_2_* absent	15	15	15
Position of *im*	Between *c3* & *la*	Between *c3* & *la*	Next to *la*	Next to *la*	Next to *lm*	Between *la* & *lm*
Position of *gla*	Next to *la*	Between La & *lm*	Anterior to *im*	Posterior to *im*	?	Anterior to *im*
Pair of epimeral ridges	**Faint**	**Faint**	Absent	Absent	Absent	Absent
Median sternal ridge	Absent	Present	Absent	Absent	Absent	Absent
Position of seta *3b*	Aligned with *4b*	Aligned with *4b*	Aligned with *4b*	Aligned with *4b*	Lateral	Lateral
*Pairs of genital setae*	3	3	3	3	3	3
*Iad* orientation	Transverse	Transverse	Transverse	Longitudinal	Transverse	Transverse
*Pairs of adanal setae*	3	3	2	2	3	2
*Pairs of anal setae*	1	1	**2**	**2**	1	1
Teeth on claws	1 Ventral	1 Ventral	1 Ventral	1 Ventral	**1 Dorsal**	**1 Dorsal**

WA = Western Atlantic, SEA = South East Africa, SJI = Southwest Japanese Islands; ? = no data available; * = alternative character state possible according to authors. Character states given in bold are unique to a specific geographic area. Data taken from literature (Grandjean 1968; Marshall and Pugh 2000; Karasawa and Aoki 2005).

Second, certain morphological traits seem to be unique for species of a specific geographic region. To be more precise, species from a specific geographic region show at least one morphological trait that is not shared by species of the other areas. The Japanese *S. nagisa* and *S. saxea* exhibit obvious lamellar ridges, laterally positioned setae *3b* and dorsal teeth on the claws, all traits unique among *Schusteria* species. The African *S. melanomerus* and *S. ugraseni* are both small and possess two pairs of anal setae instead of a single pair. The latter character is probably not restricted to a geographic area because Grandjean (1968) reported two undescribed *Schusteria* species, species B from Brazil and species C from the Pacific coast of El Salvador in Central America, both bearing two pairs of anal setae. The Western Atlantic *S. littorea* from Brazil and the Caribbean *S. marina* sp. nov. from the Lesser Antilles show short sensilli, their lyrifissures *im* are located between seta *c*_3_ and *la* and they exhibit faint epimeral ridges, all these traits either differ or lack in the other *Schusteria* species. Accordingly, there are geographic morphotypes that show slightly different morphological evolutionary trends.

The faint epimeral ridges shown in the Western Atlantic species represent an especially interesting trait. Pfingstl and Schuster (2012) already stated that the ridges of *S. littorea* may be a precursory structure of the conspicuous ventral keels shown in the genus *Carinozetes. Carinozetes* is also known to be distributed in the Western Atlantic, especially in the Caribbean area (Pfingstl and Schuster 2014; Pfingstl et al. 2016), and thus there is a clear geographical link between these taxa. Consequently, it is assumable that a *Schusteria* clade with faint epimeral ridges evolved in the Western Atlantic and that an ancestor of this clade also gave birth to the keel bearing *Carinozetes* (Figure 5). However, relationships within *Schusteria* and beyond remain largely unclear until a thorough revision and molecular genetic study of the genus and its possible relatives is performed.
